# After cochlear implantation in older adults, enhanced working memory does not fully mediate the relationship between CI and improved semantic fluency

**DOI:** 10.3389/fnagi.2025.1701934

**Published:** 2026-01-16

**Authors:** Maria Huber, Angelika Illg, Lisa Reuter, Lennart Weitgasser

**Affiliations:** 1 Department of Otorhinolaryngology, Head and Neck Surgery, Paracelsus Medical University Salzburg, Salzburg, Austria; 2 Clinic for Otorhinolaryngology, Hannover Medical School, Hannover, Germany

**Keywords:** cochlear implants, cognition, hearing loss from adulthood, semantic fluency, working memory, younger and older adults

## Abstract

**Aim:**

Studies indicate that semantic fluency improved after cochlear implantation (CI) in older adults, but not in young and middle-aged adults. We were interested in identifying cognitive variables that are associated with this improvement. We tested whether improvements in cognition after cochlear implantation are associated with improvements in semantic fluency in older adults.

**Methods:**

We used data from a multicenter cohort study. All dementia-free CI patients had symmetrical hearing loss that started in adulthood. The younger group (*n* = 20) was aged 25 and 59 years, and the older group (*n* = 21) was aged between 60 and 75 years. All participants underwent word fluency tests, as well as tests of working memory, cognitive flexibility, inhibition, and verbal episodic memory, immediately before and 12 months after the CI.

**Results:**

Semantic fluency and working memory improved significantly in the older group. No significant improvement was found in the younger group. The improvement in semantic fluency in the older group correlated significantly with the improvement in working memory. Mediation analyses suggest a partial overlap between improvements in semantic fluency and working memory. The improvement in working memory accounted for 28% of the enhancement in semantic fluency.

**Conclusion:**

In older adults with hearing loss, enhanced working memory after CI did not fully mediate the relationship between CI and semantic fluency. Additional variables that also improved after CI may influence semantic fluency.

## 1 Introduction

Word fluency refers to the ability to generate as many words as possible within a limited period of time, drawing on both crystallized and fluid intelligence (Schmidt et al., 2017). Crystallized intelligence includes semantic memory and phonological memory. Semantic memory encompasses knowledge of concepts and word meanings, while phonological memory involves information about the phonemic structure of language and letter-sound associations. Fluid intelligence includes automatic and cognitive control processes, as part of executive functioning, as well as episodic memory performance. Automatic processing primarily occurs during clustering at the beginning of a verbal fluency task. Clustering involves the production of words within a semantic or phonemic subcategory (Troyer et al., 1997). Cognitive control processes involve focused attention, working memory, cognitive flexibility, and inhibition, which are essential for controlled retrieval and word selection when continuing the verbal fluency task by shifting between clusters (Amunts et al., 2021; Biesbroek et al., 2016; Schmidt et al., 2017; Shao et al., 2014; Troyer et al., 1997; Wright et al., 2023). Episodic memory supports in sorting out repetitions and correction of erroneously produced words during retrieval (Amunts et al., 2021; Schmidt et al., 2017; Wright et al., 2023). Studies suggest that the left hemisphere of the brain, in particular, supports verbal fluency (Schmidt et al., 2019; Vonk et al., 2019; Zigiotto et al., 2022). In addition, social background influences verbal fluency performance (Mathuranath et al., 2003; Vonk et al., 2019).

Word fluency can be categorized into phonemic (letter) fluency and semantic fluency. In phonemic fluency tasks, individuals should produce as many words as possible beginning with the same letter (e.g., words starting with the letter S). In semantic word fluency tasks, individuals must generate as many words as possible that belong to the same superordinate category (e.g., animals). The processing strategies for semantic and phonemic fluency are different. Semantic fluency correlates with temporal lobe regions involved in semantic processing, including the angular gyrus, the left superior and middle temporal gyri, and the anterior part of the pars triangularis (Amunts et al., 2020; Schmidt et al., 2017). In contrast, the phonemic fluency correlates with frontal lobe regions involved in phonological processing, particularly the left inferior frontal gyrus and the pars opercularis (Konstantopoulos and Giakoumettis, 2023; Schmidt et al., 2017; Vonk et al., 2019). There is also overlap in the processing of phonemic and semantic word fluency, specifically within the frontoparietal areas responsible for word discovery and selection, speech planning, articulation planning of orofacial movements, and speech inhibition (Amunts et al., 2020; Biesbroek et al., 2016; Vonk et al., 2019). Finally, in healthy adults, phonemic fluency tasks are generally more demanding than semantic fluency tasks. A possible reason is that each word produced automatically evokes semantic associations, which must be inhibited during phonemic tasks (Vonberg et al., 2014; Woods et al., 2016).

Furthermore, verbal fluency decreases as age increases, and younger adults outperform older adults in word fluency tasks (Clark et al., 2009; Fernández et al., 2023; Gordon et al., 2018; Hoffman, 2018; Martin et al., 2023; Rodriguez-Aranda and Martinussen, 2006; Woods et al., 2016). The decline in word fluency is mainly associated with age-related reduction in cognitive control processing and memory (Burke and Shafto, 2004; Hedden and Gabrieli, 2004; Hoffman, 2018). The differences in the decline of phonemic and semantic fluency seem to be more pronounced in semantic fluency than in phonemic fluency (Clark et al., 2009; Fernández et al., 2023; Gordon et al., 2018; Hoffman, 2018; Meinzer et al., 2009; Woods et al., 2016). A possible reason for the more pronounced decline is that older adults activate the right hemisphere (the inferior and middle frontal gyri) in addition to the left hemisphere when performing semantic fluency tasks. Studies showed that additional activation is associated with lower performance (Kim et al., 2018; Meinzer et al., 2009). In contrast, in phonemic fluency tasks show no evidence of additional right hemisphere activation in either older or younger adults (Meinzer et al., 2009).

Regarding hearing loss and word fluency, mixed results are found. Stickel et al. (2024) reported a significant negative association between peripheral hearing loss (hearing thresholds >25 dB in the better ear) and phonemic fluency of middle-aged and older adults. Similarly, Samelli et al. (2022) found that each 10 dB increase in pure tone average (PTA) in the better ear of middle-aged and older adults was associated with reduced phonemic fluency. In contrast, peripheral age-related hearing loss was not significantly related to verbal fluency in older adults (Loughrey et al., 2018). Older adults with mild to severe hearing loss, many of whom wore hearing aids, did not score significantly lower in phonemic and semantic fluency tasks than their peers without hearing loss (Loughrey et al., 2020).

Additionally, with respect to possible age effects in adults with hearing loss, only one study is available. In this study, middle-aged adults with mild hearing loss scored significantly lower in phonemic and semantic fluency tasks than older adults with mild hearing loss (Chandrashekar and Nagaraj, 2024). Aside from the caveat that authors did not control the social background of the study participants, these are unexpected results, as one would expect that older adults are more affected by a decrease in phonemic and semantic fluency than young and middle-aged adults. Hearing loss-related cognitive decline is observed in old age^1^ (Deal et al., 2015; Huber et al., 2020; Loughrey et al., 2018), and verbal fluency also decreases with normal aging (see above).

Moreover, age differences in verbal fluency have also been reported in adults with hearing loss and cochlear implants (CIs). CIs are electronic medical devices that bypass the damaged hair cells in the cochlea and directly stimulate the auditory nerve (Clark et al., 1977; Hochmair and Desoyer, 1977). CI is indicated for severe and profound hearing loss when conventional hearing aids provide no or only minimal benefit (e.g., the guidelines of the Association of Scientific Medical Societies (AWMF)—(Deutsche Gesellschaft f Hals-Nasen-Ohren-Heilkunde and Kopf- und Hals-Chirurgie, 2020). CIs not only improve hearing capacity but also cognitive performance (e.g., An et al., 2023), although the latter only with minor to moderate effects (e.g., An et al., 2023). In older adults (see text footnote 1), only a partial reversal of the cognitive delay was observed 12 months after CI (Huber et al., 2021).

To date, only very few studies have investigated the effect of CIs on semantic fluency. Völter et al. (2018) reported a significant improvement in older adults 12 months after CI. However, the authors did not provide specific information on the performance of phonemic and semantic fluency, and they also did not correct for multiple comparisons. Another study investigated older adults (60–75 years) and young and middle-aged adults (25–59 years) with severe and profound hearing loss from adulthood (Huber et al., 2025). Twelve months after CI, semantic fluency had significantly improved in the older group when compared to the younger group. Phonemic fluency did not improve in either the older or the younger group (Huber et al., 2025).

Apart from the need for further studies to corroborate these findings (Huber et al., 2025), we were interested in exploring the reasons for this improvement. Our question was whether cognitive variables that improved after CI affect semantic fluency in older adults. This study investigated whether positive changes in cognitive performance could serve as possible mediators between CI and semantic fluency.

## 2 Methods

In this cohort study on semantic fluency and cognition of CI patients, data from Huber et al. (2025) were used. This study was part of a project on the depressive and cognitive status, as well as verbal fluency of younger and older adults before cochlear implantation and 12 months after CI **(**Huber et al., 2023, 2025). Data were collected between 2019 and 2024 at two tertiary reference centers. The local ethics committees granted ethical approval (protocol numbers 8419_BO_S_2019 and 415-E/22489/2-2019).

### 2.1 Participants

A total of 61 participants had signed the informed consent form for the first CI. For study participation, the participants had to fulfill the following *inclusion criteria*: age between 25 and 75 years, symmetrical sensorineural hearing loss (the difference in hearing thresholds between the two ears was no more than 20 dB), and onset of hearing loss from adulthood (>18 years), and the four-frequency PTA on the contralateral ear was at least 40 dB hearing loss. *Exclusion criteria* included unilateral deafness, sudden sensorineural hearing loss in the last 12 months before CI surgery, retro-cochlear hearing loss, blindness, regular use of anticholinergics, previous and/or current systemic treatment of a malignancy, current psychotic illness, or a current affective disorder with psychotic symptoms, poor German language skills, and a very low non-verbal IQ (< 80), corresponding to a range of less than 5% in the Matrices Test (Petermann and Lepach, 2012; Wechsler, 2012). Twelve months after surgery, the study population had decreased to 41 participants. The selection process for the study and reasons for non-participation are reported elsewhere (Huber et al., 2023, 2025). Table 1 presents demographic, audiometric, and clinical information for the younger group (aged 25 to 59 years, *n* = 20) and the older group (aged 60 to 75 years, *n* = 21) before CI. The results of the audiometric testing after CI are reported in Huber et al. (2025). No changes were observed after 12 months in comorbidities (diabetes and arterial hypertension) and in unhealthy behaviors (smoking).

**Table 1:** Demographic, audiometric, educational, and clinical data of young and middle-aged adults (younger group) and older adults (older group).

**Age, years mean (SD)**	**Younger group (25–59 years, *n* = 20)**	**Older group (60–75 years, *n* = 21)**	**Comparison *p***
	51.15 (10.12)	67.76 (5.51)	< 0.001
**Sex, No. (%)**			0.64
Female	9	11	
Male	11	10	
**Education level** ^a^ **, No. (%)**			0.90
1	11 (61%)	9 (64%)	
2	5 (28%)	3 (21%)	
3	2 (11%)	2 (15%)	
Educational years, mean (SD)	14.25 (2.31)	12.90 (3.59)	0.08
**Audiometric results (PTA dB HL), Median (min–max)**			
CI ear	80.00 (65.00–110.00)	88.75 (61.25–120.00)	0.21
Other ear	63.75 (28.75–110.00)	76.25 (28.75–120.00)	0.08
**Present risk factor, No. (%)**			
Nicotine use current	2 (10%)	2 (10%)	0.96
Nicotine use previous	6 (30%)	4 (19%)	0.41
Arterial hypertension	6 (30%)	9 (43%)	0.46
Diabetes mellitus type 1	0	0	1.00
Diabetes mellitus type 2	1 (5%)	2 (10%)	0.61
Hypercholesterolemia	3 (15%)	4 (19%)	0.79
**COVID disease during study period, No**.			0.06
Yes	8 (50%)	13 (81%)	
No	8 (50%)	3 (19%)	
**Cause of hearing loss** ^a^ **, No. of patients (%)**			0.51
Sudden hearing loss	5 (25%)	4 (19%)	
Otitis media	0	1 (5%)	
Noise	0	3 (14%)	
Trauma	0	0	
Middle ear surgery	2 (10%)	2 (10%)	
Genetic	0	0	
Unknown	9 (45%)	8 (38%)	
Other	4 (20%)	3 (14%)	
**Duration of hearing loss** ^a^ **, Years, mean (SD)**			
Years, mean (SD)	17.30 (11.19)	21.90 (13.92)	0.03
**Use of hearing aids**^a^**, No**.			
General, No. of patients	20	20	
Bilateral, No. of patients	19	17	0.17
No use, No. of patients	0	1	0.32
**Duration of hearing aid use** ^a^ **, Years, mean (SD)**			
Left ear	13.07 (10.33)	12.24 (9.50)	0.79
Right ear	13.57 (9.90)	12.90 (10.80)	0.84
**Speech recognition monosyllables with HA in %, mean (SD)**			
CI ear^b^	30.00 (28.28)	18.27 (26.04)	0.25
Other ear	50.83 (31.35)	26.43 (28.31)	0.19

Characteristics of hearing loss and data on hearing aid usage of participants at the time of first investigation (before cochlear implantation).

No., Number; SD, Standard deviation; PTA, Pure tone average (0.5–4 kHz); dB, Decibel; HL, Hearing level, HA, Hearing aid. 1 = Secondary school/middle school/junior high, 2 = Ordinary level/high school, 3 = University.

^a^Information of the patient.

^b^Ear on which the cochlear implantation should be performed.

### 2.2 Measurement and instruments

#### 2.2.1 Primary outcome

The mediator quality of positive change scores in the older group 12 months after CI was examined in cognitive control processing (working memory, cognitive flexibility, and inhibition) and verbal episodic memory, with respect to the effect of CI on semantic fluency. Change scores are the difference in performance after CI compared to before CI.

### 2.3 Assessment of hearing status

Hearing function was assessed by trained audiologists in double-walled, soundproof booths in accordance with the International Electrotechnical Commission (IEC) regulations. Pure tone thresholds, whereas higher thresholds (four-frequency PTA) indicated lower hearing status (Table 1).

Speech recognition in quiet was assessed using the Freiburg Monosyllables test (FMT), which consists of 20 recorded monosyllabic words per test series (Hesse et al., 2009; Lehnhardt, 2001). The test was performed in a free-field measurement at 65 dB sound pressure level (SPL). The percentage of correctly perceived words was documented (Table 1). Prior to CI, the patients wore their hearing aids with the programming they had received from their local acoustician (Table 1). During testing after cochlear implantation, the patient's wore their CI. Before and after CI, tests were conducted exclusively on the CI side to monitor the success of the surgery, as specified in the routine clinical protocols. The contralateral ear was masked when indicated, following the standard procedures (Studebaker, 1964; see also Lenarz et al., 2012).

### 2.4 Assessment of verbal fluency, cognitive control, and episodic memory

Age- and education-adjusted standard scores from the cognitive tests enabled control of age-related cognitive decline (see Introduction).

The Regensburg Word Test (RWT) was used to test phonemic and semantic word fluency (Aschenbrenner et al., 2000; Baldo et al., 2006). The task is to produce as many unique words as possible that begin with a specific letter (e.g., S, phonemic version) or belong to a given category (e.g., fruit, semantic version) at a given time. The number of correctly produced words was recorded.

The computerized n-back test (Test of Attentional Performance (TAP) by Zimmermann and Fimm (2019)) was used to evaluate attention and working memory, reflecting both the control of information flow (active maintenance) and the ability to update information continuously (Cohen et al., 1997; Kirchner, 1958; Maguire et al., 2009). In this test, numbers appear on the display in sequence. The task is to click the mouse to determine whether the number is identical to a previously shown number. The number of omissions was documented.

To test cognitive flexibility, the Trail Making Test-B (TMT-B) was used (Cohen et al., 1997; Giovagnoli et al., 1996; Reitan, 1958). In this paper-based test, numbers and letters must be connected alternately in ascending order as fast as possible. We documented the reaction time using the standard scores of Rodewald et al. (2012).

Furthermore, the Go/NoGo Test of the TAP was used to evaluate focused attention and inhibition (Maguire et al., 2009). The task of this test is to suppress a response triggered by an external stimulus in favor of an internally controlled behavioral response. The reaction time was noted.

The Verbal Learning and Memory Test (VLMT) was used to assess episodic verbal memory (Helmstaedter and Lendt, 2001; Schmidt, 1996). The VLMT measures various parameters of episodic verbal memory, such as immediate recall and delayed recall after 30 min. This study focused on immediate recall because the results of a previous study indicate that CI patients have specific problems with learning tasks (Huber et al., 2020). In VLMT learning, the investigator presented fifteen unrelated nouns orally in five rounds. The investigator recorded the number of correctly reproduced words per round.

The VLMT consists of auditory (orally presented) tasks, which implies that hearing loss can affect the results of this test. Despite potential biases, we have decided to use this test. The VLMT is the only validated test of verbal episodic memory in German, suitable for young, middle-aged, and older adults (Helmstaedter and Lendt, 2001). We mentioned the use of this test as a limitation of our study in the discussion.

All test instructions were provided both orally and in writing. All cognitive tests were visual (non-auditory) except for the VLMT. Communication during test administration had to follow a standard template. Clinical psychologists and clinical staff experienced in interaction with individuals with hearing loss conducted the cognitive tests. These staff received additional training and were supervised by clinical psychologists, as TAP tests may only be administered by psychologists (see TAP manual).

### 2.5 Statistical analysis

For statistical analysis, SPSS 29.02.0 and R 4.4.1 were used. Comparisons between pre- and post-CI performance measures were conducted by paired sample *t*-tests and mixed ANOVAs in SPSS 29.02.0. To identify potentially mediators of significant change scores in semantic fluency, we correlated the significant change scores in cognitive performance with those in semantic fluency (change scores defined as the differences between pre- and post-CI scores). To account for the small sample size and violations of normality in some cognitive variables, non-parametric Spearman correlations were used. Because there is evidence that cognition improves after CI (see above) and only improvements in cognitive scores are relevant to the research question, one-sided significance tests are used. Multiple comparisons were corrected using the false discovery rate (FDR). To address whether increases in semantic fluency performance were mediated by cognitive control or episodic memory, mediation analyses were conducted using the *mediation* package in R 4.4.1. To obtain more robust estimates due to the small sample size, bootstrapped mediation was used. Thereby, CI (pre/post CI) was used as the independent variable, semantic fluency as the dependent variable and cognitive control or episodic memory as potential mediators. The percent change in variance explained by the independent variable was assessed after accounting for the mediator.

## 3 Results

### 3.1 Changes in semantic fluency and cognitive performance in younger and older groups 12 months after CI

As described in Huber et al. (2025) and above, older, but not younger, groups showed significant improvements in semantic fluency after CI. Twelve months after surgery, the older adults produced an average of 3.71 [95% CI = [1.65; 5.78]] words more. Likewise, older, but not younger, groups showed significant improvements in verbal working memory, assessed using the n-back task (Table 2). However, the improvement in working memory was not significantly more pronounced in older adults than in younger adults. No significant changes in cognitive flexibility, inhibition, and episodic memory in either group were observed (Table 2).

**Table 2:** Changes in semantic fluency, cognitive control processing, and episodic memory 12 months after CI, compared to the situation before CI (younger group of 25–59 years, *n* = 20; older group of 60–75 years, *n* = 21).

	≤ **60 years**							***≥*** **60 years**							**Comparison**	
	**Pre-CI**		**12 months CI**			**Change**		**Pre-CI**		**12 months CI**			**Change**		**Time** ^*^ **group**	
	**Mean**	**SD**	**Mean**	**SD**	**Mean**	**SD**	***T***	**Mean**	**SD**	**Mean**	**SD**	**Mean**	**SD**	***T***	η^2^*_*p*_*	***F***
**Verbal fluency**																
RWT semantic [PR]	43.80	27.81	39.15	30.45	−4.65	27.88	−0.75	**53.33**	**34.66**	**72.71**	**26.24**	**19.38**	**26.20**	**−3.39** ^***^	**0.17**	**8.09** ^**^
**Executive functions**																
N–back [T]	53.40	11.57	53.35	10.78	−0.05	13.33	0.02	**44.79**	**16.36**	**55.80**	**14.16**	**10.28**	**17.74**	**−2.46** ^*^	0.10	4.17
TMT-B [PR]	22.50	25.31	30.00	26.95	7.50	16.50	−2.03	19.05	18.95	22.86	23.27	3.81	18.57	−0.94	0.01	0.45
Go/NoGo [T]	47.80	11.37	45.65	9.70	−2.15	12.21	0.78	50.00	9.64	50.33	9.10	0.05	11.61	−0.02	0.01	0.34
**Episodic memory**																
VLMT learning [T]	52.65	8.77	57.10	9.23	4.45	8.59	−2.32	49.00	11.42	50.57	10.37	1.57	8.09	−0.89	0.03	1.22

RWT, Regensburg Word Test; N-back, Working memory; TMT-B, Trail Making Test-B, cognitive flexibility; Go/NoGo, Inhibition; VLMT, Verbal Learning and Memory Test, verbal episodic memory, immediate recall; T, T Score; PR, Percentile rank; SD, Standard deviation; Rows in bold font show significant effects (*p*-value corrected for multiple comparisons using false discovery rate) * p_FDR_ < 0.05; ** p_FDR_ < 0.01; *** p_FDR_ < 0.001.

### 3.2 Associations between significant positive change scores in working memory and semantic fluency in the older group

In older adults, changes in semantic fluency were significantly positively associated with changes in working memory (n-back) [*r* = 0.54 [0.07; 0.81], *p* = 0.02]. Greater the improvements in working memory after CI, the higher the increase in semantic fluency.

### 3.3 Mediation analysis (older group)

**Figure 1:**
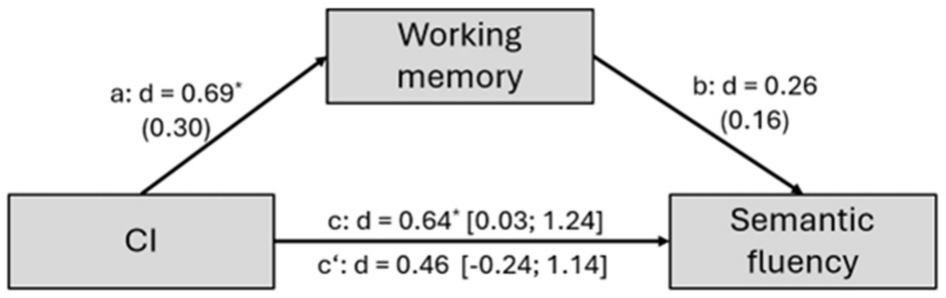
Results of mediation analyses. Mediation of cochlear implants (CI) effects on semantic fluency by working memory. CI, Independent variable; Semantic fluency, Dependent variable; Working memory, Potential mediator. Direct effect: C'/C = 0.46/0.64 = 0.72; Indirect effect: a × b = 0.69 × 0.26 = 0.18; ^*^ *p* < 0.05.

In the older group, the changes in working memory as potential mediators of the changes in semantic fluency were evaluated. Although both CI implantation and working memory were significantly related to semantic fluency, neither remained significant when both variables were included in a multiple regression model. Working memory changes accounted for 28% of changes in semantic fluency performance, indicating a 28% overlap in explained variance (Direct effect: C'/C = 0.46/0.64 = 0.72. see Figure 1). However, the indirect effect [*d* = 0.18 [−0.03; 0.54], *p* = 0.11] was non-significant, suggesting an ineffective mediation pathway (see Figure 1).

## 4 Discussion

We found that in older adults, improvement in semantic fluency correlated with enhanced working memory after CI. However, working memory did not fully mediate the effect of CI on semantic fluency. There was only an overlap in explained variance, suggesting that additional factors beyond working memory may influence semantic fluency after CI.

We presume that social participation is one possible candidate. Social participation is “a person's involvement in activities that provide interaction with others in society or the community” (Levasseur et al., 2010). In adults without hearing loss, social participation is associated with both phonemic and semantic fluency (Rehan and Phillips, 2025) and with working memory performance (Kelly et al., 2017). Older adults with hearing loss struggle to maintain relationships and participate in social activities (Prieur Chaintré et al., 2024). Social participation improves after CI (Issing et al., 2024; Mertens et al., 2021; Nijmeijer et al., 2021).

All improvements in working memory and semantic fluency occurred without any training in verbal fluency or cognitive abilities, and the improvement in semantic fluency of approximately 3.7 words is probably clinically relevant. For orientation, patients with a probable Alzheimer's (AD) diagnosis produced an average of two words less than the healthy subjects in both tasks. Patients with an AD diagnosis produced an average of three fewer words in each task (Olmos-Villaseñor et al., 2023).

Regrettably, we had no control group of peers without hearing loss. Therefore, it is not possible to determine whether a decline in semantic fluency existed before CI, and if so, whether there was a reversal of the decline after CI. These are essential questions, because deterioration in semantic fluency is recognized as a risk factor for dementia (Gustavson et al., 2020).

We have already mentioned some limitations of this study. A further limitation is the small sample size. As discussed in the Method section, auditory cognitive tests, like the VLMT, can bias the results. However, the study also has some strengths. To our knowledge, these are the first findings on possible correlations between changes in cognitive control, memory, and semantic fluency in adults with hearing loss after CI. Furthermore, the probability of bias in the study is estimated to be low.

## 5 Summary and conclusion

Following cochlear implantation in older adults, improvements in semantic fluency were significantly associated with enhanced working memory. However, working memory did not fully mediate the relationship between CI and semantic fluency. Additional positive changes after a CI, which are also related to changes in working memory, for example, in social participation, may potentially affect semantic fluency.

## Data Availability

The data analyzed in this study is subject to the following licenses/restrictions: We used data from a project on the depressive and cognitive status of CI-patients (Huber et al., 2025, Ear and Hearing, in print). Requests to access these datasets should be directed to M.Huber@salk.at.

## Footnotes

1 There are no studies with this aim, addressing young and middle-aged adults.
